# The Importance of Age in the Prediction of Mortality by a Frailty Index: A Machine Learning Approach in the Irish Longitudinal Study on Ageing

**DOI:** 10.3390/geriatrics6030084

**Published:** 2021-08-27

**Authors:** Sebastian Moguilner, Silvin P. Knight, James R. C. Davis, Aisling M. O’Halloran, Rose Anne Kenny, Roman Romero-Ortuno

**Affiliations:** 1 Nuclear Medicine School Foundation (FUESMEN), National Commission of Atomic Energy, Mendoza M5500CJI, Argentina; moguilns@tcd.ie; 2 The Global Brain Health Institute (GBHI), Trinity College Dublin, D02 PN40 Dublin, Ireland; 3 The Irish Longitudinal Study on Ageing (TILDA), Trinity College Dublin, D02 R590 Dublin, Ireland; silvin.knight@tcd.ie (S.P.K.); davisj5@tcd.ie (J.R.C.D.); aiohallo@tcd.ie (A.M.O.); rkenny@tcd.ie (R.A.K.)

**Keywords:** frailty, age distribution, longitudinal studies, mortality, supervised machine learning, sex differences

## Abstract

The quantification of biological age in humans is an important scientific endeavor in the face of ageing populations. The frailty index (FI) methodology is based on the accumulation of health deficits and captures variations in health status within individuals of the same age. The aims of this study were to assess whether the addition of age to an FI improves its mortality prediction and whether the associations of the individual FI items differ in strength. We utilized data from The Irish Longitudinal Study on Ageing to conduct, by sex, machine learning analyses of the ability of a 32-item FI to predict 8-year mortality in 8174 wave 1 participants aged 50 or more years. By wave 5, 559 men and 492 women had died. In the absence of age, the FI was an acceptable predictor of mortality with AUCs of 0.7. When age was included, AUCs improved to 0.8 in men and 0.9 in women. After age, deficits related to physical function and self-rated health tended to have higher importance scores. Not all FI variables seemed equally relevant to predict mortality, and age was by far the most relevant feature. Chronological age should remain an important consideration when interpreting the prognostic significance of an FI.

## 1. Introduction

As populations get older, the association between chronological age and health status becomes increasingly heterogeneous [1]. To describe this heterogeneity in health status as we age, the concepts of biological age [2] or frailty versus fitness spectrum [3] have been proposed. The frailty index (FI) methodology was introduced by Rockwood and colleagues [4,5] to quantify the accumulation of people’s health ‘deficits’ (i.e., symptoms, clinical signs, medical conditions and disabilities) at a given chronological age. This method has allowed for the establishment of potentially useful population norms [6] and the study of influences of wider determinants of health on the variation in health status within people of a similar chronological age [7]. Since FI deficits increase with age [8], the FI has a statistically significant association with chronological age [9]. However, on the account of population heterogeneity, the effect size of this association has been found to be small [10,11]. It has been suggested that given the age-related nature of its constituent deficits, the FI should be interpreted jointly with age [12].

Previous work has shown that women have higher FI scores than men at all ages [13]. The FI has been found to be a significant predictor of mortality [14] and a limit to deficit accumulation has been demonstrated at around 0.7 [15]. However, whilst women tend to accumulate more deficits, their risk of mortality tends to be lower [4]. These important sex differences have prompted many researchers to report FI associations separately by sex [16,17].

The FI is a count of deficits [5] and presumes that the number of things that are wrong is more important than what is wrong [18,19]. In a busy clinical setting, time may not be available to measure all FI components; as such, it would be useful to know which features may be more prognostically important and therefore should be looked at and addressed first.

Our aim was to utilize data from The Irish Longitudinal Study on Ageing (TILDA) to conduct, separately by sex, supervised machine learning analyses of the ability of the individual items of an FI to predict 8-year mortality. To gain insights as to the importance of age in this prediction, we repeated the analyses including age as a feature.

## 2. Materials and Methods

### 2.1. Design and Setting

We analyzed data from TILDA, a population-based longitudinal study of ageing. Wave 1 of the study (baseline) took place between 2009 and 2011, and subsequent data were collected approximately twice yearly over four subsequent longitudinal waves (wave 2: 2012–2013; wave 3: 2014–2015; wave 4: 2016; wave 5: 2018). The full cohort profile has been described previously [20].

### 2.2. Measures

#### 2.2.1. Construction of the Frailty Index (FI)

A 32-item FI was constructed using self-reported health measures recorded at wave 1 of TILDA [21]. The selection of deficits was consistent with the standard FI requirements [8], including that deficits are any symptom, sign, disease, or disability associated with age and adverse outcomes, are present in at least 1% of the population, cover several organ systems and have under 5% missing data [21]. The components of this 32-item FI, and an individual item scoring scheme, are shown in Appendix A. Previous work has suggested that FI variables can be dichotomous or ordinal, with little impact on the predictive ability of the FI [22].

#### 2.2.2. Mortality Data

Mortality was ascertained for all study participants at each follow-up wave from Ireland’s Central Register Office [23].

### 2.3. Descriptive Analyses

Descriptive statistics were computed with IBM SPSS Statistics version 25 (IBM Corp., Armonk, NY, USA) and given as mean with standard deviation (SD) and range, or proportion (%). The association between the FI and age was measured with the 2-sided Spearman’s correlation coefficient (*r*_s_) and quadratic R^2^.

### 2.4. Machine Learning (ML) Analyses

ML analyses were conducted separately for men and women to investigate the ability of the 32 FI items at baseline to predict subsequent 8-year mortality. To investigate the importance of age vis-à-vis FI items, two ML analyses were conducted for each sex: one with age included as a feature, and one without age. To avoid the potential problem of class imbalance, the samples were balanced prior to each ML analysis by randomly sampling participants from the non-deceased sub-cohort with similar numbers to the deceased. 

For ML analyses, we split the datasets in a ratio of 80% for training, and 20% for testing, using random division, to test for generalizability without employing the testing dataset during the validation phase. Feature variables were normalized using the min-max method. For the training phase, we employed a 5-fold cross-validation for hyper-parameter tuning. We employed a linear discriminant analysis (LDA), a widely used supervised ML classifier algorithm. Some advantages of the LDA are its computationally fast implementation and its easy adaptation for discriminating non-linearly separable classes, through the kernel trick method [24]. Classification accuracy values were accompanied by: (i) calculations of the area under the curve (AUC) of the receiver operating characteristic (ROC) curve; (ii) confusion matrices capturing the sensitivity and specificity of each classification; and (iii) a complete ranked feature importance list for each classification. To obtain unbiased results for our analyses when employing imbalanced datasets, we reported both micro-averaged and macro-averaged AUC scores. The former pools the scores across classes, and then computes the overall AUC score. The latter just computes a simple average of the AUC scores over classes. Results were reported on the 20% testing samples.

### 2.5. Ethics

Ethical approval for each wave was obtained from the Faculty of Health Sciences Research Ethics Committee at Trinity College Dublin, Ireland: Wave 1: “The Irish Longitudinal Study on Ageing (granted 2 May 2008)”; Wave 2: “The Irish Longitudinal Study on Ageing (granted 19 October 2011)”; Wave 3: “Main Wave 3 Tilda Study (granted 9 June 2014)”; Wave 4: “Ref: 150506”; and Wave 5: “Ref: 170304”. All participants provided written informed consent.

## 3. Results

In TILDA wave 1, there were 8174 participants aged 50 or more (mean (SD) age 63.8 (9.8) years, range (50–105)), and 54.2% were women. In the total sample, the mean FI for men was 0.11 (SD 0.10) and 0.13 (SD 0.11) for women. None of the 32 FI items had any missing data. The histograms of the FI by sex are shown in Appendix B. The associations between FI and age were of moderate effect size, both in men (*r*_s_ = 0.39, *p* < 0.001, N = 3744) and women (*r*_s_ = 0.41, *p* < 0.001, N = 4430). The quadratic R^2^ between age and the FI was 0.14 in men and 0.17 in women.

By wave 5, 559 men (14.9%) and 492 women (11.1%) had been recorded as deceased. From the non-deceased cohort, 566 men and 494 women were randomly selected to provide the balanced samples for the ML analyses. The age and FI characteristics of these four groups vis-à-vis those of the entire cohort are presented in Table 1. The total ML analytical samples were 1125 men and 986 women.

**Figure 1:**
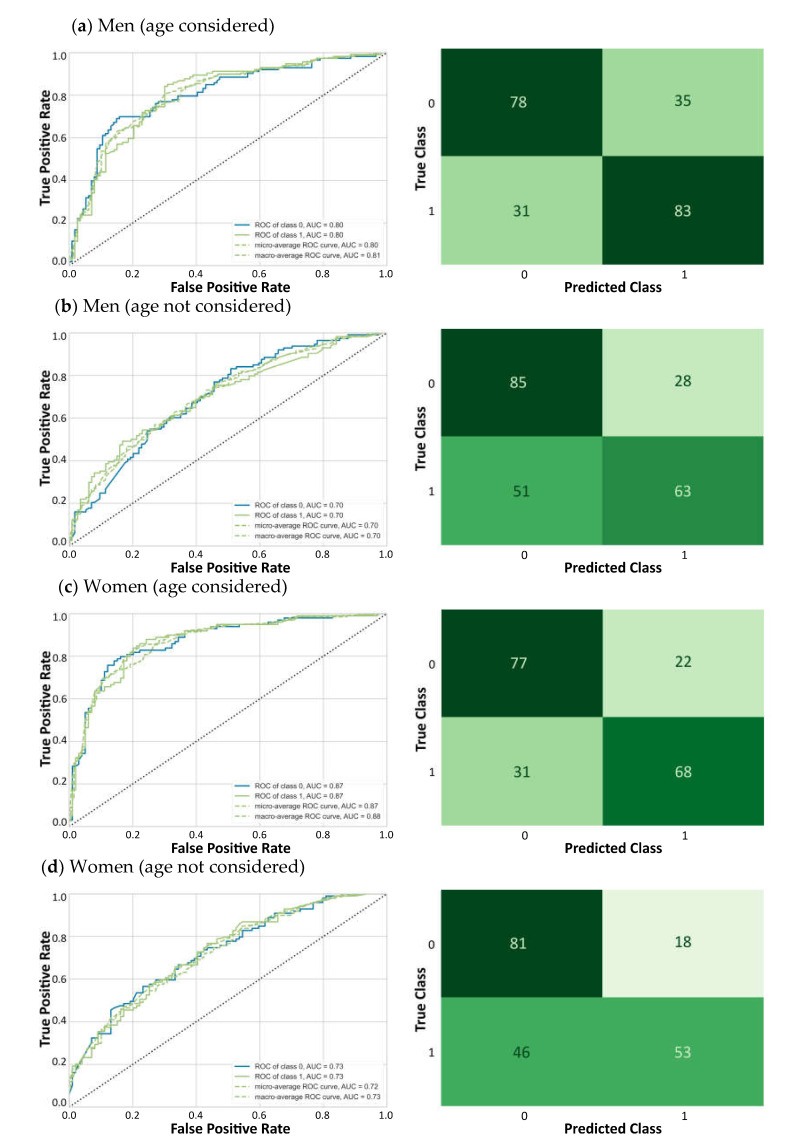
ROC curves and confusion matrices.

**Figure 2:**
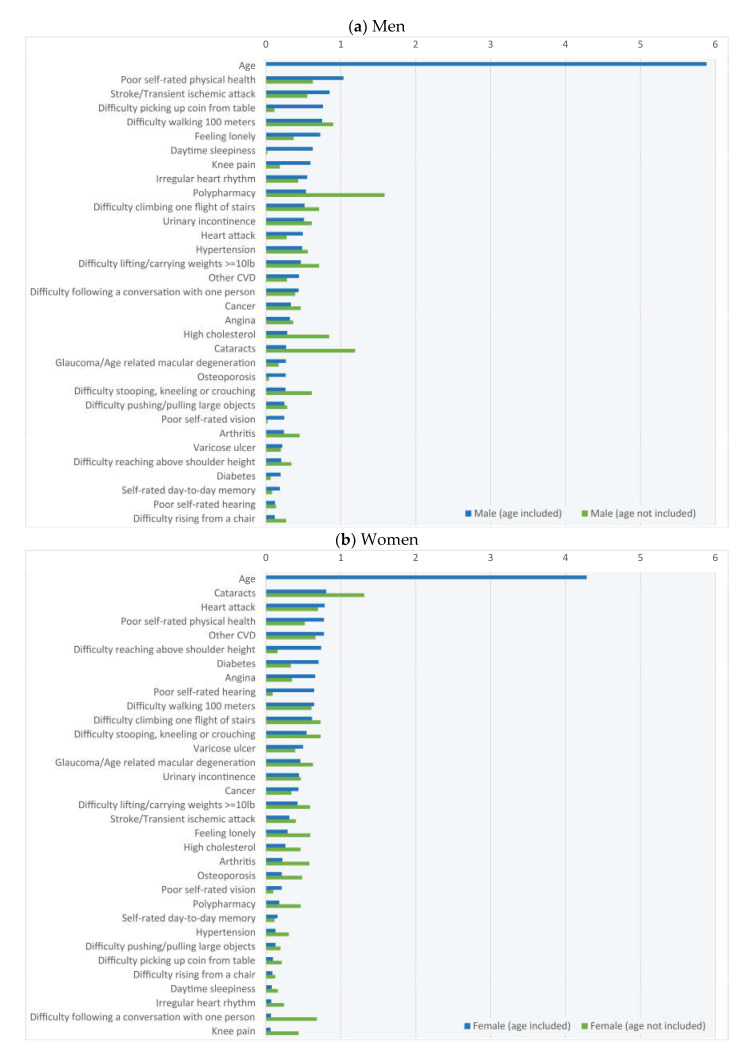
Frailty index feature importance.

The ROCs, AUCs and confusion matrices for each classification on the 20% testing samples are presented in Figure 1. Male panels (a) and (b) (N = 227) show results with age included and not included, respectively; and panels (c) and (d) show the same for women (N = 198). Figure 2 shows, for men (a) and women (b), the predictor importance for the classifications when including (blue) and not including (green) age. When age was included, this was by far the most important predictor in both men (importance score: 5.9) and women (4.3).

## 4. Discussion

Our study revealed that in the absence of age, the FI was an acceptable predictor of mortality with AUCs of 0.7. When age was included, AUCs were improved to values of 0.8 in men and 0.9 in women. Even in a balanced samples design, ML models seemed best at classifying true negatives (i.e., non-deceased), and the addition of age seemed to improve their performance to classify true positives (i.e., deceased participants). In terms of the feature importance, when age was included, it stood out as the most important feature vis-à-vis the FI items. Outside of these large age effects, there was a comparatively narrower degree of variation in the importance of the other FI items. The inclusion of age in the models led to some changes in the ranking of the top 10 FI features. Overall, our results suggest that the addition of chronological age significantly enhanced the ability of the FI to classify mortality events. 

On average, deceased subgroups were 11 years older than alive subgroups, whereas the difference in baseline FI was about 0.1 (Table 1). In the context of a 32-item FI, the latter is equivalent to say that, on average, people who had died after 8 years had, compared to those who remained alive, three more FI deficits and were more than a decade older. Indeed, the passing of a decade is of high physiological and clinical relevance, with well documented evidence as to how the function of multiple physiological systems declines per every decade of life starting in early adulthood [25]. In community samples, FI studies have suggested that deficits accumulate at an average rate of approximately 3% per annum on a log scale [5]. In a large sample of Europeans [10], it was found that the average FI for men in their 60s was 0.10, increasing to 0.14 (0.14–0.15) in their 70s; whilst for women, it was 0.13 (0.12–0.13) in their 60s and 0.18 (0.18–0.19) in their 70s. In a normative values study utilizing CLSA data [6], the 50th percentile FI for men in their 60s was 0.07 (0.06–0.08), increasing to 0.10 (0.08–0.11) in their 70s; whilst for women, it was 0.08 (0.07–0.09) in their 60s and 0.11 (0.09–0.12) in their 70s. Given this, and as suggested in Table 1, our deceased subgroups seemed to have, in the context of their chronological age group, higher-than-expected FI values, which mirror the ML results that the FI was relevant for mortality prediction independently of age. Even though both age and FI were relevant in predicting mortality, the effect of age was much more pronounced, which is not surprising given that FI deficits are chosen to be age-related. Our results are in line with a previous theoretical proposition [12] that proposed that the consideration of the FI score together with chronological age may be more informative for the prediction of clinical outcomes than the consideration of the FI alone.

In an early study by Rockwood’s group, an artificial neural network (ANN) of an FI based on self-reported deficits was found to be superior to the unweighted FI in predicting survival in older Canadians, with an ROC curve of 86% for the ANN and 62% for the FI [26]. In keeping with our sex-split design and findings, they noted that with age and sex excluded as predictors in the model, the percentage of correctly classified persons reduced to 80%. Additionally, in keeping with our results, they found that the prediction of survival for women showed higher accuracy than that of men [26].

In our ML models, outside of the large age effects, the importance of other individual FI items was comparatively narrower, with importance scores between 0 and 1. This may suggest small clinical effect differences between the individual FI items; however, this may not necessarily be in keeping with the theoretical tenet that ‘the number of things that are wrong matters more than what is wrong’ [5,18,19]. For example, an exploratory factor analysis on an FI in CLSA showed that the strongest underlying factor had high loadings from physical functional status and self-rated health; the second, from life satisfaction; and the third, from depressive symptoms including loneliness [11]. Their findings, together with the previous suggestion that the combination of FI variables underlying survival might be different in men and women [26], resonate with our results.

Indeed, in our age-included ML models, the second most important feature in men was poor self-rated physical health, and three in the top 10 referred to physical function difficulties (i.e., picking up a coin, walking 100 m) and loneliness. The other top 10 referred to specific morbidities (stroke/TIA, irregular heart rhythm), symptoms (sleepiness, knee pain) and polypharmacy. As regards sex differences in health outcomes associated to poor self-reported health, it has been suggested that it may better reflect the risk of mortality in men than in women, to the extent that clinicians may need to take the poor self-rated health of older men even more seriously [27]. As regards self-reported joint/musculoskeletal pain, women are more likely to report widespread pain; however, men are more likely to show objective radiographic changes [28]. Analogous to this may be the fact that whilst loneliness shows a tendency to be associated with all-cause mortality in both sexes, the effect may be slightly higher in men [29]. On the other hand, it has been suggested that daytime sleepiness may be more linked with obstructive sleep apnea in men and depression in women [30], with the former generally carrying more adverse cardiovascular mortality implications. The association of polypharmacy with mortality has been reported to be stronger in men [31].

In women (age-included model), the second most important feature was cataracts. Poor self-rated physical health and physical function difficulties (i.e., reaching above shoulder height, walking 100 m) were also in the top 10 predictors. Other top 10 predictors included cardiovascular disease (i.e., heart attack, other cardiovascular disease, angina), diabetes and poor hearing. As regards age-related cataracts, it has been described as a predictor of poorer survival and a possible marker of frailty [32]. A study showed that the mortality associated with cataracts was higher in women [33]. However, the literature on sex differences in mortality associated with cataracts is scarce, and the clinical plausibility of a higher mortality risk in women is not immediately obvious. On the other hand, it is well known that more than in men, cardiovascular mortality in women accelerates from the age of 60 [34]. The relative risk of fatal cardiovascular disease associated with diabetes is higher in women than men [35]. Interestingly, hearing loss could be a marker of underlying cardiovascular disease [36].

Strengths of our study include the large initial sample size, a long (8-year) follow-up for the collection of mortality events and an FI that fulfilled standard properties in terms of the minimum number of deficits and their requirements [8], distribution (Appendix B), association with age (quadratic R^2^ = 0.15), higher values in women and a higher limit at 0.7 (Table 1). 

However, our study also has important limitations. Despite the large initial sample size, the 20% testing samples on which our results are based were comparatively small. The fact that the FI was constructed on self-reported (as opposed to objective) health measures may be a limitation; however, FI scholars have argued that measuring self-rated health by an index of deficits is a valid approach [37]. The 32 deficits in our FI were ‘manually’ pre-selected as per hypothesis-based standard procedure [8], but others have used a data-driven approach to deficits selection using ML methods that automatically select variables based on the best fitness of the model [38]. 

Another possible limitation of our design is that the collection of 8-year mortality as a dichotomous outcome does not consider the exact time to death within that period, and predictors of short-term mortality may be different than those of longer-term mortality. A much larger sample would have been needed to implement a more nuanced short- versus long-term mortality approach. In our study, we employed random training (80%) and testing (20%) divisions, and 5-fold cross-validation procedures for hyper-parameter tuning, but these do not necessarily eliminate the possibility of overfitting, which is a frequent occurrence in ML analyses. Our findings have not been replicated in a separate cohort, and hence are not necessarily generalizable to other populations. Thus, in terms of the relative importance of the FI items, and despite the clinical plausibility of many, we cannot recommend that the FI items that we identified as most important are the ones that clinicians should necessarily prioritize. Even from a statistical perspective, given the underlying assumptions of LDA (i.e., normal distribution of independent variables and equal variance–covariance matrices within each group), and even though all variables were normalized prior to analyses, we can be more confident about the classification predictions (i.e., the significant added effect of age) than the analysis of the relative importance of features. Indeed, from a clinical management point of view, a comprehensive geriatric assessment (and hence attention to all deficits present in an individual) remains the gold standard to assess and address the complex problems that older adults present to clinical services [39]. However, at a population level, ML-based learnings could be used as a base for developing decision-support tools to improve early identification and prediction of at-risk older adults, or to monitor disease patterns to inform policy design.

## 5. Conclusions

In conclusion, the addition of chronological age significantly enhanced the ability of a 32-item self-reported FI to classify mortality events. In clinical practice, it may be more informative to refer to an FI score in the context of the person’s chronological age. Results from the ML analysis performed herein would argue against the theoretical tenet that ‘the number of things that are wrong matters more than what is wrong’. However, our findings are not necessarily generalizable and replication on external samples is required.

## Figures and Tables

**Table 1:** Age and FI characteristics of the four balanced groups vis-à-vis those of the entire cohort.

Characteristic	Men Not Deceased N = 566	Men Deceased N = 559	Women Not Deceased N = 494	Women Deceased N = 492	Entire Cohort 45.8% Male N = 8174
Age (years)	62.7 (SD: 8.5 (50–89))	73.6 (SD: 9.6 (50–96))	63.4 (SD: 9.4 (50–90))	74.7 (SD: 10.8 (50–105))	63.8 (SD: 9.8 (50–105))
Difficulty walking 100 m	3.2% (N = 18)	18.2% (N = 102)	6.3% (N = 31)	26.6% (N = 131)	7.4% (N = 601)
Difficulty rising from a chair	11.0% (N = 62)	22.9% (N = 128)	13.8% (N = 68)	30.3% (N = 149)	18.0% (N = 1470)
Difficulty climbing one flight of stairs	4.2% (N = 24)	18.4% (N = 103)	5.7% (N = 28)	25.6% (N = 126)	7.7% (N = 633)
Difficulty stooping, kneeling, or crouching	16.8% (N = 95)	37.4% (N = 209)	19.6% (N = 97)	50.2% (N = 247)	27.7% (N = 2262)
Difficulty reaching above shoulder height	4.2% (N = 24)	12.5% (N = 70)	6.7% (N = 33)	16.5% (N = 81)	8.0% (N = 651)
Difficulty pushing/pulling large objects	6.5% (N = 37)	19.5% (N = 109)	13.4% (N = 66)	33.7% (N = 166)	12.7% (N = 1041)
Difficulty lifting/carrying weights ≥ 10 pounds	8.1% (N = 46)	25.0% (N = 140)	20.0% (N = 99)	46.1% (N = 227)	18.3% (N = 1496)
Difficulty picking up a coin from a table	3.2% (N = 18)	8.8% (N = 49)	3.9% (N = 19)	11.4% (N = 56)	4.1% (N = 331)
Feeling lonely (0.5/1)	2.8/0.9% (N = 16/5)	5.7/3.4% (N = 32/19)	5.3/1.6% (N = 26/8)	9.6/4.7% (N = 47/23)	5.2/2.1% (N = 425/174)
Poor self-rated physical health (0.5/1)	19.4/3.4% (N = 110/19)	29.3/12.0% (N = 164/67)	16.6/4.7% (N = 82/23)	30.7/13.0% (N = 151/64)	18.2/5.1% (N= 1484/417)
Poor self-rated vision (0.5/1)	9.7/1.4% (N = 55/8)	11.4/3.2% (N = 64/18)	9.7/1.4% (N = 48/7)	14.4/5.7% (N = 71/28)	8.1/1.6% (N = 663/131)
Poor self-rated hearing (0.5/1)	13.6/3.4% (N = 77/19)	22.4/3.9% (N = 125/22)	11.3/1.4% (N = 56/7)	14.8/4.5% (N = 73/22)	11.8/2.4% (N = 962/194)
Self-rated day-to-day memory (0.5/1)	14.7/3.7% (N = 83/21)	20.0/6.3% (N = 112/35)	17.2/1.8% (N = 85/9)	18.7/6.5% (N = 92/32)	13.5/2.9% (N = 1102/233)
Difficulty following conversation with 1 (0.5/1)	7.6/0.7% (N = 43/4)	14.1/2.1% (N = 79/12)	3.4/0.8% (N = 17/4)	10.6/2.0% (N = 52/10)	5.7/0.8% (N = 469 /67)
Daytime sleepiness (0.5/1)	16.6/18.7% (N = 94/106)	20.9/23.1% (N = 117/129)	9.5/13.0% (N = 47/64)	13.2/22.8% (N = 65/112)	15.1/14.9% (N = 1230/1216)
Polypharmacy	14.8% (N = 84)	40.6% (N = 227)	18.8% (N = 93)	40.2% (N = 198)	20.8% (N = 1682)
Knee pain	4.2% (N = 24)	7.0% (N = 39)	7.3% (N = 36)	13.2% (N = 65)	7.6% (N = 621)
Hypertension	30.7% (N = 174)	44.0% (N = 246)	34.4% (N = 170)	48.8% (N = 240)	37.1% (N = 3031)
Angina	5.0% (N = 28)	13.4% (N = 75)	1.8% (N = 9)	10.6% (N = 52)	5.5% (N = 449)
Heart attack	8.1% (N = 46)	14.7% (N = 82)	1.0% (N = 5)	7.5% (N = 37)	4.6% (N = 378)
Diabetes	9.0% (N = 51)	13.1% (N = 73)	5.5% (N = 27)	12.0% (N = 59)	7.8% (N = 634)
Stroke/Transient ischemic attack	2.3% (N = 13)	8.6% (N = 48)	2.8% (N = 14)	8.9% (N = 44)	3.6% (N = 291)
High cholesterol	33.6% (N = 190)	30.1% (N = 168)	35.2% (N = 174)	35.8% (N = 176)	38.1% (N = 3111)
Irregular heart rhythm	5.3% (N = 30)	12.9% (N = 72)	5.3% (N = 26)	11.6% (N = 57)	7.2% (N = 588)
Other cardiovascular disease	3.0% (N = 17)	6.6% (N = 37)	0.8% (N = 4)	5.5% (N = 27)	3.6% (N = 294)
Cataracts	6.7% (N = 38)	22.9% (N = 128)	9.7% (N = 48)	30.9% (N = 152)	10.9% (N = 889)
Glaucoma/Age-related macular degeneration	2.7% (N = 15)	5.7% (N = 32)	2.6% (N = 13)	7.9% (N = 39)	4.0% (N = 325)
Arthritis	18.1% (N = 103)	27.7% (N = 155)	26.1% (N = 129)	46.3% (N = 229)	27.6% (N = 2255)
Osteoporosis	1.6% (N = 9)	2.7% (N = 15)	12.8% (N = 63)	20.1% (N = 99)	9.6% (N = 786)
Cancer	5.7% (N = 32)	10.0% (N = 56)	7.1% (N = 35)	12.8% (N = 63)	6.3% (N = 512)
Varicose ulcer	1.6% (N = 9)	3.9% (N = 22)	2.4% (N = 12)	8.1% (N = 40)	3.3% (N = 271)
Urinary incontinence (0.5/1)	1.9/2.5% (N = 11/14)	3.2/10.0% (N = 18/56)	5.9/14.2% (N = 29/70)	5.5/17.7% (N = 27/87)	3.2/9.4% (N = 259/765)
TOTAL—Frailty Index Score	0.09 (SD: 0.09 (0–0.55))	0.17 (SD: 0.13 (0–0.70))	0.11 (SD: 0.10 (0–0.52))	0.22 (SD: 0.14 (0–0.69))	0.12 (SD: 0.11 (0–0.70))

**Figure A1:**
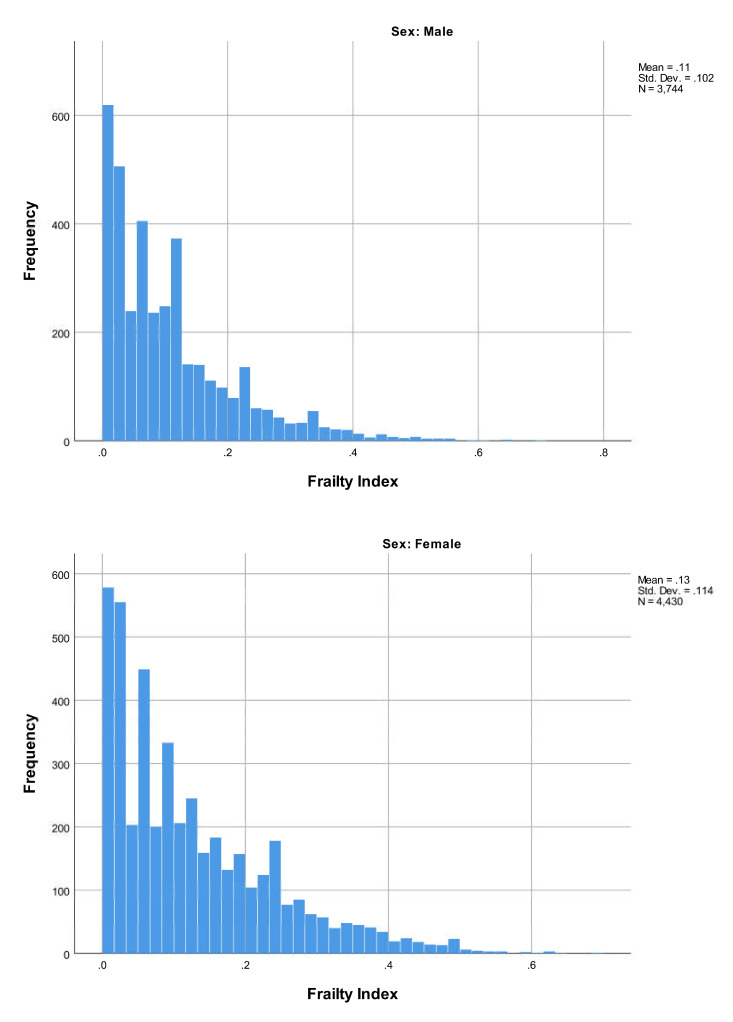
Distribution of the FI in men and women (total sample).

## Data Availability

The data underlying the results cannot be shared due to ethical and data protection issues. Requests to access this data can be made directly to TILDA (tilda@tcd.ie) and will be considered on a case-by-case basis. The first four waves of TILDA data are available from the Irish Social Science Data Archive (ISSDA) at www.ucd.ie/issda/data/tilda/ (accessed on 2 July 2021). To access the TLDA survey data, please complete an ISSDA Data Request Form for Research Purposes, sign it and send it to ISSDA by email (issda@ucd.ie).

## Appendix A

**Table A1:** 32-item frailty index: items and scoring of individual items.

Self-reported deficit	Scoring
Difficulty walking 100 m	0 = No; 1 = Yes
Difficulty rising from a chair	0 = No; 1 = Yes
Difficulty climbing one flight of stairs	0 = No; 1 = Yes
Difficulty stooping, kneeling or crouching	0 = No; 1 = Yes
Difficulty reaching above shoulder height	0 = No; 1 = Yes
Difficulty pushing/pulling large objects	0 = No; 1 = Yes
Difficulty lifting/carrying weights ≥ 10 pounds (4.5 Kg)	0 = No; 1 = Yes
Difficulty picking up a coin from a table	0 = No; 1 = Yes
Feeling lonely	0 = None of the time, rarely; 0.5 = Some of the time; 1 = All the time
Self-rated physical health	0 = Excellent, Very good, Good; 0.5 = Fair; 1 = Poor
Self-rated vision	0 = Excellent, Very good, Good; 0.5 = Fair; 1 = Poor
Self-rated hearing	0 = Excellent, Very good, Good; 0.5 = Fair; 1 = Poor
Self-rated day-to-day memory	0 = Excellent, Very good, Good; 0.5 = Fair; 1 = Poor
Difficulty following a conversation with one person	0 = None; 0.5 = Some; 1 = Much/Impossible
Daytime sleepiness	0 = Never, slight chance; 0.5 = Moderate chance; 1 = High chance
Polypharmacy	0 = No; 1 = Yes
Knee pain	0 = No; 1 = Yes
Hypertension	0 = No; 1 = Yes
Angina	0 = No; 1 = Yes
Heart attack	0 = No; 1 = Yes
Diabetes	0 = No; 1 = Yes
Stroke or Transient ischemic attack	0 = No; 1 = Yes
High cholesterol	0 = No; 1 = Yes
Irregular heart rhythm	0 = No; 1 = Yes
Other cardiovascular disease	0 = No; 1 = Yes
Cataracts	0 = No; 1 = Yes
Glaucoma or Age-related macular degeneration	0 = No; 1 = Yes
Arthritis	0 = No; 1 = Yes
Osteoporosis	0 = No; 1 = Yes
Cancer	0 = No; 1 = Yes
Varicose ulcer	0 = No; 1 = Yes
Urinary incontinence	0 = Never, slight chance; 0.5 = Moderate chance; 1 = High chance
